# Responses to the updated Nutri-Score algorithms in Norway: A qualitative study among food system actors in the NewTools-project

**DOI:** 10.29219/fnr.v68.10254

**Published:** 2024-10-01

**Authors:** Mari Mohn Paulsen, Bente Øvrebø, Anne Lene Løvhaug, Kaja Lund-Iversen, Lene Frost Andersen, Arnfinn Helleve, Marianne Hope Abel

**Affiliations:** 1 Department of Food Safety, Norwegian Institute of Public Health, Oslo, Norway; 2 Centre for Sustainable Diets, Norwegian Institute of Public Health, Oslo, Norway; 3 Centre for Evaluation of Public Health Measures, Norwegian Institute of Public Health, Oslo, Norway; 4 Department of Nursing and Health Promotion, OsloMet – Oslo Metropolitan University, Oslo, Norway; 5 Research Administrative Support, Norwegian Institute of Public Health, Oslo, Norway; 6 Department of Nutrition, Institute of Basic Medical Sciences, University of Oslo, Oslo, Norway; 7 Department of Physical Health and Ageing, Norwegian Institute of Public Health, Oslo, Norway

**Keywords:** nutrient profiling, stakeholder responses, front-of-pack nutrition label, food system

## Abstract

**Background:**

Nutri-Score is a front-of-pack label grading foods and beverages from A to E indicating nutritional quality based on the foods’ favorable and unfavorable components, and a contender in the ongoing debate on the possible implementation of a harmonized mandatory front-of-pack nutrition label in the European Union. NewTools is a research project on scoring systems for foods involving 28 partners representing actors involved in the Norwegian food system.

**Objective:**

This study aimed to explore views reported by Norwegian food system actors on the advantages and disadvantages with the updated Nutri-Score algorithms for food and beverages (2022–2023). This included Nutri-Score’s performance in ranking foods according to the national food-based dietary guidelines and to the nutritional challenges in Norway.

**Design:**

A total of 28 project partners and 15 other food system stakeholders following the NewTools-project were invited to provide responses on the Nutri-Score algorithms and their application on foods and beverages in the Norwegian food composition table. Thirteen written responses were received and analyzed with qualitative content analysis.

**Results:**

The responses to the updated Nutri-Score varied in content, reflecting mainly concerns. Examples of perceived concerns included excessive penalty of salt content; insufficient differentiation based on fat content in meat, sausages, cheese, and milk; and several unreasonable comparisons across food categories. They also expressed a concern that Nutri-Score may stimulate to increased food processing, and some reported inconsistencies between Nutri-Score’s classification of foods and national nutrition guidelines and policies.

**Discussion and conclusion:**

Several concerns with the updated Nutri-Score algorithms were raised, including the weighting of specific nutrients, unfair outcomes when comparing across food categories, and inconsistencies with established Norwegian nutrition guidelines and policies. The results should be interpreted with caution, as some perspectives from the Norwegian food system may be missing.

## Popular scientific summary

- Nutri-Score is a front-of-pack label grading foods and beverages according to nutritional quality.
- This paper presents responses from actors involved in the Norwegian food system to the updated Nutri-Score algorithms.
- Several food system actors expressed specific concerns with the Nutri-Score algorithms, particularly related to salt and saturated fat. Inconsistency with established Norwegian nutrition policies was also raised as a concern.
- The responses contribute with insight and add useful knowledge when evaluating Nutri-Scores’ alignment with the Norwegian dietary guidelines.

A suboptimal diet is a major contributor to increased risk of non-communicable diseases and morbidity (1), as well as higher healthcare costs (2). Promoting healthy diets is important for public health (3), and nutrition labeling policies are ways governments might improve population diets (4). A front-of-pack nutrition label (FOPNL) can be a tool in aiding consumers to choose healthier foods by providing nutritional information in a simplified way to the consumer and by stimulating healthier product development (5). The European Commission proposed to launch a harmonized mandatory FOPNL for the European Union (6), and the Nutri-Score label has been one of the debated contenders (7, 8).

Nutrient profiling systems underpin FOPNLs (4), and Nutri-Score’s underlying algorithms use favorable and unfavorable components in foods to grade them on a five-color scale associated with letters from A, indicating higher nutritional quality to, E, indicating lower nutritional quality (9). The Scientific Committee of Nutri-Score published updates for the Nutri-Score algorithms for foods in 2022 (10) and for beverages in 2023 (11) (hereinafter together referred to as the updated Nutri-Score). The update aimed to improve several limitations that have previously been identified (12–14), and the updated Nutri-Score is reported to align more with food-based dietary guidelines in the European countries engaged in Nutri-Score (10, 15).

France, Belgium, Germany, Spain, Luxemburg, the Netherlands, and Switzerland have currently implemented Nutri-Score as a voluntary FOPNL (16), but the implementation has been challenging. In France, multiple lobbying strategies were deployed to stop or at least delay the implementation of Nutri-Score (17). Several countries and various actors within the food system have opposed the use of the Nutri-Score, referring to limitations associated with its reliance on grams or milliliters rather than actual portion sizes, and because it is said to favor artificial foods over natural foods (18) and punish local and cultural food (19, 20). Opposing arguments from various actors in the food system are addressed in the media or public reports, but there is, to our knowledge, no scientific research aiming to objectively present food system actors’ responses to the updated Nutri-Score.

NewTools is a 4-year research project involving 28 partners representing sectors and organizations with different roles and mandates in the Norwegian food system, such as research institutions, food production and industry, retail, governmental agencies, and non-governmental organizations (21). The project is also open to other stakeholders in the food system (referred to as other stakeholders following the project in this paper), who receive newsletters, can participate in seminars, and can be invited to contribute if relevant. To date, additional 15 stakeholders representing civil society, food industry, research institutions, government agencies, and health service have asked to receive information and to be involved where possible in the project. The primary aim of the NewTools-project is to contribute to the development of two scoring systems for foods: one for nutritional quality and one for environmental- and social sustainability, and a secondary aim is to explore their potential areas of application with partners in the project.

A recent evaluation of the updated Nutri-Score in a Norwegian context concluded with an overall satisfactory discriminatory performance and alignment with the Norwegian food-based dietary guidelines (22). However, a few inconsistencies were reported such as inability to discriminate between regular and reduced-fat alternatives of cheese, cooking creams, and sausages, and between whole grain and refined pasta and rice (22). To capture more of the potential strengths and weaknesses related to the updated Nutri-Score and further potential needs for revisions, it is also relevant to consult actors in the food system and voice their views on implications for foods and food production, processing, and manufacturing food system actors.

The aim of this study was to explore views reported by Norwegian food system actors on the advantages and disadvantages of the updated Nutri-Score algorithms for foods (2022) and beverages (2023). This included Nutri-Score’s performance in ranking foods and beverages, according to the food-based dietary guidelines and to the nutritional challenges in Norway.

## Methods

### Study design and setting

A qualitative approach was used to explore views reported by food system actors to the updated Nutri-Score in a Norwegian context. Both partners in and stakeholders following the NewTools-project were invited purposively through e-mail to provide their responses through two consultations. We calculated the updated Nutri-Score on the food and beverages (hereinafter referred to as foods) included in the publicly available Norwegian food composition table (23), which made the basis for the consultation. Nutri-Score was presented in total points and class (A–E) for each food in an Excel-file, and in tables and figures showing the distribution of Nutri-Score for main- and subcategories of foods. Relevant materials shared in the consultations are found in the supplemental material A–D and have been translated from Norwegian to English for publishing purposes. The first consultation covered responses to the updated Nutri-Score algorithm for foods (2022), and the second consultation covered the updated beverage algorithm (2023). We followed the Standards for Reporting Qualitative Research (24) and the Consolidated criteria for Reporting Qualitative research (COREQ) checklists (Supplemental material E) in reporting our findings. Since the present study utilized written submissions instead of interviews or focus groups, not all COREQ items were applicable.

### Data

All partners and stakeholders following the NewTools-project were invited to participate. This approach was used as the NewTools-project, involves actors across the Norwegian food system covering all major food groups and who are committed to contribute with information. The responses had to be written and signed by the invited partner or stakeholder following the project, in accordance with the framework for engagement in the NewTools-project (25). Responses were collected by e-mail. The written responses were considered as representing the institutional view and not the individuals who signed. A total of 28 project partners and 15 stakeholders following the project (hereinafter together referred to as food system actors unless specified) were invited by e-mail.

Actors involved in NewTools were invited by the researchers to attend two digital information meetings where information about the consultations on the updated Nutri-Score and its calculation was provided. The information given in the meetings was also provided to all actors by e-mail in connection with the information meetings. The aims of the consultations were presented, as well as figures showing the distribution of Nutri-Score for various food groups and for beverages in the second consultation. In total, 24 food system actors participated in the meeting about the food algorithm and 19 participated in the meeting about the beverage algorithm. A recording of the presentations during the meetings was distributed afterward, along with a link to the recordings. However, we did not track who or how many viewed the recordings.

The actors were asked to give their responses on how the updated Nutri-Score algorithms would perform in Norway considering the food-based dietary guidelines (32) and the dietary challenges in the population. They were specifically asked to assess challenges with ranking of foods, and not challenges related to the use of such a ranking to, for example, guide consumers. They were encouraged to elaborate and use examples in their responses and were invited to comment on any topics they found relevant, but the following questions were listed as suggestions for relevant aspects to consider in both consultations:

- Are there any products/categories/ingredients that are given a Nutri-Score considered too low or too high?
- Does Nutri-Score adequately distinguish between healthier and less healthy products within logical product categories (products with similar use)?
- Are there any components that are given too much or too little weight in the algorithm?
- Are there any important dimensions not captured in the Nutri-Score algorithm?
- Are there any areas of mismatch between Nutri-Score and the Norwegian food-based dietary guidelines?
- Are there any advantages or disadvantages related to the fact that Nutri-Score is primarily based on available data on the packaging and could it be appropriate to include other components, for example, whole grains, added sugar, proportion of fish, etc., like the Keyhole, which is a voluntary Nordic label for food (26), does?

### Data analysis

The food system actors could formulate their responses freely, and the written responses varied from one to seven A4 pages. To map the responses, we conducted a basic qualitative content analysis (27, 28), aiming to describe and categorize the responses. The software NVivo version 14 was used to facilitate the analysis.

First, the responses were read by five of the authors (BØ, MMP, ALL, KLI, and MHA), all of whom hold a PhD or an MS in nutrition and were researchers in the NewTools-project, to get an overall overview of the material. Second, data were organized into categories sharing similar content. The organization of categories was reviewed in several iterations through discussions between the first and second authors and finally organized into a structure of categories and subcategories that has been used to present the findings. Three of the researchers (MMP, ALL, and AH) had experience and training in qualitative research methods. Researcher reflexivity was also considered, taking into account the researchers’ roles within the field of nutrition and public health, as well as their involvement in the NewTools-project on scoring systems.

Participant validation (29) was used by inviting all actors who contributed with written submissions to provide feedback on a draft of the article and the interpretation of their responses. This included how their written responses were presented and analyzed in the article. This was done due to the NewTools-project being a collaborative project with an established framework for stakeholder engagement (25). Information about the possibility to give feedback was distributed by e-mail with a deadline of two weeks. Of all actors, five submitted responses to the draft. We revised the manuscript to reflect their feedback for accurate representation of their specific responses or quotations. Responses introducing new information or additional perspectives not included in the initial responses were not incorporated into the manuscript. We took into account suggestions regarding the portrayal of food system actors in the discussion section and made adjustments based on feedback, indicating that several actors from the food industry focused mainly on their own products rather than on the entire range of products available on the Norwegian food market.

Trustworthiness in the analysis (30) was emphasized. This included credibility by inviting actors representing different parts of the food system to submit responses; dependability by involving several authors in the categorization of the results and using a software program to help sort, organize, and code the material; and using participant validation (29).

### Ethics

A written information letter was distributed in the consultation rounds, specifying that the responses provided could be used in a scientific article. All written submissions were treated as institutional, and no personal data were used in the consultations.

## Results

We received a total of 12 written responses in the first consultation for the food algorithm and one written submission in the second consultation for the beverage algorithm. The responses from the food industry actors primarily focused on their own products. A significant proportion (70%) of the invited actors refrained from responding in the consultations. Table 1 shows the submissions, food system sector, and food group/field of interest.

**Table 1:** Submissions to the updated Nutri-Score from food system actors in the NewTools-project

Submission no.	Food system sector	Food group/primary field of interest
1	Civil society organization	Consumers
2	Food industry^1^	Meat
3	Food industry^1^	Meat
4	Food industry^1^	Meat
5	Food industry^1^	Primary production and farmers
6	Food industry^1^	Grain
7	Food industry^1^	Grain
8	Food industry^1^	Dairy
9	Food industry^1^	Dairy and beverages^2^
10	Food industry^1^	Ready meals and meal components
11	Food industry^1^	Oils/margarines, spreads, and ready meals
12	Research institution	Education and research institution
13	Food industry^1^	Association for food manufacturers

1 The ‘food industry’ includes primary producers, food manufacturers, retailers, and organizations representing these actors.

2 Include juice, plant-based beverages, and other beverages.

**Fig. 1:**
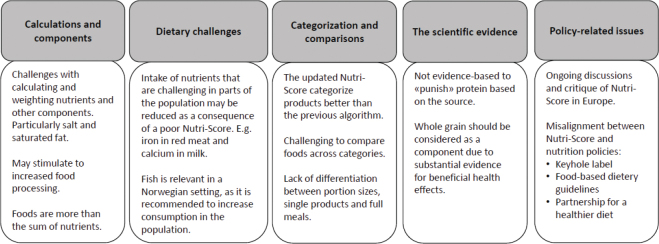
Overview of the main categories (grey boxes, top row) and responses to the updated Nutri-Score in a Norwegian context from actors in the NewTools-project.

Only responses related to the Nutri-Score algorithms (*n* = 12) are presented in this article, as the final response concerned more overarching aspects of the NewTools-project. Content from the 12 written submissions was grouped into five main categories reflecting different forms of arguments. The results in each main category are summarized in Fig. 1, and a more comprehensive overview of responses to calculations and components is given in Table 2.

**Table 2:** Responses to calculations and components in the Nutri-Score algorithms

Component or aspect of Nutri-Score	Quote (submission number)	Food system sector providing the response
**Fat**		
Lack of differentiation between full-fat and reduced fat alternatives for sausages, meat, cheese, and milk.	‘*There is a poor distinction between original and leaner products. For example, original sausages and lean sausages receive the same score.’* (sub no. 5)	Primary production and farming*
	‘*All white milks, except whole-fat milk, receive a B. For the consumer, this will appear as if you can just as well choose a milk with 1.8% fat as a fat-free milk.*’ (sub no. 9)	Dairy sector and beverages*
The saturated fat component works well in practice for ready meals.	*‘The algorithm for saturated fat seems to function well in practice.’* (sub no. 10)	Producer of ready meals and meal components*

**Fiber and whole grain**		

Whole grain should be included in the food algorithm, rather than fiber.	‘*We question whether it is appropriate for fiber to stand as a separate positive criterion. In line with dietary recommendations, the intake of fiber should preferably be increased through increased intake of whole grains, fruit, vegetables, and legumes. […] A condition on fiber alone will lead to many launches [of products] with added fiber, and we question whether this is desirable both in a sustainability and nutrition perspective*.’ (sub no. 8)	Dairy sector*
Nutri-Score does not differentiate between degree of coarseness in flour.	‘*We also do not understand why white wheat flour, white spelt flour and refined baking flour receive the score A, just as good as whole wheat flour.*’ (sub no. 12)	Education and research institution*
The fiber component in the food algorithm should have a wider interval.	‘*It is good that the fiber scale has been extended […]. Still, the scale could go even higher.’* (sub no. 6)	Grain sector*
The fiber component in the food algorithm does not work well for ready meals.	*‘The fiber algorithm does in practice not affect ready meals. […] In a Norwegian context one would think that oat or barley porridge receives a better score than rice porridge when the recipes only include milk, grain and a little salt.’* (sub no. 10)	Producer of ready meals and meal components*

**Salt**		

Too strict salt intervals in the algorithms.	‘*The limits for salt in Nutri-Score should be re-considered. A product can contain a very low content of salt to receive a low score in this area. The Norwegian population consumes more salt than the health authorities recommend. The level nevertheless appears unreasonably low in relation to the Norwegian nutrient recommendations, and thus not sufficiently justified in terms of health.*’ (sub no. 2)	Meat sector. *Grain sector. Primary production and farmers. Producer of ready meals. Producer of various food products. Education and research institution.
The salt limits may compromise food safety.	*‘The ‘penalty’ for adding salt is so high that it can also compromise food safety (some salt is necessary for food processing).’* (sub no. 4)	Meat sector*. Primary production and farmers.

**Sugar and artificial sweeteners**		

Disagreement with the definition of added sugar.	*‘There is some disagreement around the definition of added sugar. In some cases, it may be useful to differentiate between whole fruits and pureed fruits. This may apply, for example, to products for children and fruit smoothies.’* (sub no. 1)	Consumer organization*
Added versus naturally occurring sugar in milk products.	*‘A potential nutrition scoring system should be based on added sugar. Sugar naturally present in milk is not a nutritional concern, and sugar content should not be used as a proxy for added/free sugar.’* (sub no. 8)	Dairy sector*
Products receiving a poor score even though they are nutritious for children.	*‘Both regular and soft whey cheeses receive an E. These products are excellent sources of iodine and are also a good choice as a spread in line with dietary guidelines for schools and kindergartens.’* (sub no. 8)	Dairy sector*
Non-nutrients, including artificial sweeteners.	*‘The score does not take into account “non-nutrients”, such as sweeteners. We believe that the possibility of creating products within different categories that appears healthy regardless of the use of sweeteners may lead to greater exposure to products sweetened with artificial sweeteners.’* (sub no. 8)	Dairy sector*
The sugar component does not affect ready meals.	*‘The sugar component does not affect ready meals in practice. The interval between the points is too large. […] However, as sugars are not a nutritional challenge in this product category it may not have a very large impact.’* (sub no. 10)	Producer of ready meals and meal components*

**Protein**		

The protein cap for meat is too strict, affecting nutrient dense and healthy foods.	*‘The consequence of how Nutri-Score is currently calculated is that nutrient dense, unprocessed foods of red meat are portrayed as unhealthy: The majority of unprocessed red meat and other red meat products obtain a score of D or E […].’* (sub no. 2)	Meat sector*
The protein cap is positive as we should limit red meat consumption.	*‘We support the limited number of protein points for red meat, as it is desirable for the population to reduce its intake of red meat.’* (sub no. 1)	Consumer organization*
The protein cap does not work for ready meals.	*‘It is positive that changes have been made for protein so that red meat receives a maximum of 2 points and protein is weighted positively for fish, but this does not seem to have an impact on ready meals in practice.’* (sub no. 10)	Producer of ready meals and meal components*

**Vitamins and minerals**		

Products should not obtain favorable points for adding vitamins not naturally occurring in the product.	*‘It is important that added vitamins, which are not vitamins that the population is deficient in, are not rewarded with a higher score.’* (sub no. 1)	Consumer organization*
Vitamins and minerals are not taken into consideration for red meat.	*‘It is also difficult to understand […] why one does not include other criteria such as different vitamins and iron.’* (sub no. 5)	Primary production and farmers*. Meat sector

**Energy**		

The food algorithm does not discriminate between the energy density of ready meals.	*‘The interval is too wide […]. The algorithm is therefore not a good tool to discriminate between less and more energy dense dishes.’* (sub no. 10)	Producer of ready meals and meal components*

**Fruit, vegetable, and legumes**		

The cut-off of 40% content of fruit, vegetables, or legumes is too high for ready meals.	*‘The fruit, vegetables, and legumes component does not have a practical impact on ready meals. […] More than 40% vegetables are challenging in many types of ready meals because vegetables contribute to a lot of liquid runoff. […] For comparison, the criteria for fruit, vegetables, and legumes are 28% in the Keyhole Regulations. The criterion of >40% vegetables limits the ability to navigate the category effectively.’ (*sub no. *10)*	Producer of ready meals and meal components*

**Fish**		

Fish should be included as a favorable component.	*‘There should be specific requirements for different food categories. The proportion of fish in fish products is an example of this. […] As it is desirable for the population to consume more fish, this is something that is especially relevant in a Norwegian context.’* (sub no. 1)	Consumer organization*

**Milk**		

Milk should be considered as a favorable component.	*‘Could points for the proportion of milk have solved the challenge that milk’s role as a contributor of important nutrients in the diet seems to be somewhat blurred in the algorithm?’* (sub no. 9)	Dairy sector and beverages*

**Processing**		

Nutri-Score may stimulate more processed foods.	*‘Nutri-Score may stimulate increased processing, based on the criteria set for a favorable score. Highly processed foods that are only made from added ingredients (no whole food ingredients) and with limited nutritional value are rewarded with a good score, for example some plant-based meat substitutes. […] There are many examples of the Nutri-Score algorithm favorizing heavily processed and partly nutrient-poor products.’* (sub no. 4)	Meat sector* Dairy sector
Ultra-processing may be included in the future, but there is currently not enough evidence.	*‘We have noticed that there has been some discussion around the degree of food processing. The most important aspect is that Nutri-Score is based on scientifically grounded knowledge, and it is somewhat unclear whether ultra-processing can be included at this time. However, it is possible that it might be relevant in the future.’* (sub no. 1)	Consumer organization*

**Food matrix effects**		

Nutri-Score does not take the natural interaction of substances in foods into consideration	*‘Because the algorithm primarily focuses on nutrients, it does not take into account the fact that nutrients and non-nutrients in foods interact and contribute to a health effect that cannot be attributed to the sum of nutrients alone.’* (sub no. 8)	Dairy sector*

**Weighting of components**		

Challenging to understand how the different components are weighted.	‘It is timely to ask why Nutri-Score allows a relatively higher sugar content compared to saturated fat, even though the recommended intake for both nutrients is <10 percent of the total energy intake. The intake of saturated fat and sugar in the population has about the same deviation from the recommended intake.’ (sub no. 2)	Meat sector*.
		Primary production and farming
There should be a more even distribution of foods along the A to F scale of Nutri-Score.	‘For any potential nutritional scoring system to contribute to healthier choices within a category, there should be a more even distribution across the A to E scale.’ (sub no. 8)	Dairy sector*

* Actor providing the quote. Actors listed without asterisk provided a response related to the specific component/aspect, but this response is not quoted.

Keyhole label: Voluntary Nordic label for foods (26), Partnership for a healthier diet: Collaboration between the food industry and the health authorities to improve the diet of the population (31).

### Responses to calculations and components in the algorithms

Many of the food system actors raised concerns about the calculations of specific nutrients and food components in the updated Nutri-Score algorithms. This included energy; saturated fat; sugar; artificial sweeteners; salt; proteins; fiber; and fruit, vegetables, and legumes. They also raised concerns about components or dimensions that are not currently part of the updated Nutri-Score, including whole grain, vitamins and minerals, specific product groups such as fish and milk, and food processing level. The weighting of the different components in the Nutri-Score algorithms was also questioned. These responses are summarized below and described in detail in Table 2.

#### Reported concerns related to calculation of components

Food system actors reported that the updated Nutri-Score does not effectively differentiate between full-fat and reduced-fat alternatives of foods and beverages. Examples cited included sandwich meat toppings, sausages, cheese, and milk. Food system actors upheld that the scale for fiber in the algorithm should be able to cover a larger interval. Questions were also raised as to why Nutri-Score does not differentiate between degree of coarseness in flour. Many of the responses were related to concerns that the salt component of Nutri-Score receives too much weight in the food algorithm. These claimed that salt intervals were too strict, resulting in healthy products obtaining a poor score. Food system actors discussed the definition of sugar and what should be taken into consideration in the algorithms, as Nutri-Score calculation currently is based on total sugars. Artificial sweeteners are not taken into account in the updated Nutri-Score algorithm for foods (10), and a concern was raised whether this may lead to a higher exposure for artificially sweetened products in the population. Red meat has a cap on number of favorable points from protein in the updated Nutri-Score food algorithm (10). This cap was criticized as being too strict, thus limiting a wider distribution in score for red meat and meat products. The protein cap was, however, supported by another actor due to the dietary recommendation of limiting red meat. In addition, this actor supported that fish should be able to receive a higher score than red meat. It was also highlighted that Nutri-Score does not capture meaningful variation in nutritional quality of ready meals.

#### Missing components in the updated Nutri-Score algorithms

In the updated Nutri-Score algorithms, vitamins and minerals are not directly included (10). Food system actors pointed to the content of important micronutrients in meat and dairy products. One actor claimed that a product should not obtain favorable points for adding vitamins not naturally occurring in the product, however, that it may be considered in cases of government-initiated fortification.

The importance of food matrix effects was emphasized in one response, which reported that foods are more than the sum of their individual nutrients. According to this actor, the Nutri-Score system fails to account for the natural interplay of substances within foods.

Nutri-Score was criticized for not taking degree of processing into consideration in the algorithm. Actors claimed that Nutri-Score may stimulate increased processing based on the criteria set for a favorable score, and that there are many examples of Nutri-Score favoring heavily processed products. One actor suggested that ultra-processing of foods may be included in the score in the future, but that it must be evidence-based.

### Dietary challenges in the population

A concern raised was related to whether a limited intake of red meat, due to poorer Nutri-Score for red meat and meat products, may increase the risk of iron deficiency among women in reproductive age. A similar concern was raised for iodine and calcium in milk and dairy products.

*‘Considering the challenging iodine and calcium situation in Norway, it is a challenge if nutrient poor drinks are seen as equally good alternatives to fat-free fermented milk or full-fat milk.’* (Submission no. 9, Dairy sector)

Only one response was received to the updated beverage algorithm. This response asserted that Nutri-Score may introduce an argument to skip milk in favor of artificially sweetened soft drinks or other nutrient-poor drinks that do not make a positive contribution to the diet. Other reported concerns were that fruit juice appears to be as unhealthy as soft drinks, and that soy-based beverages appear to be as healthy as milk.

One food system actor pointed to fish as a food of special relevance in a Norwegian setting, as it is recommended that the population increase their intake of fish and fish products and suggested that Nutri-Score should have specific requirements to take this into account.

### Categorization of products and comparisons across food categories

Food system actors raised a concern related to difficulties in comparing foods across food categories. This issue was particularly raised for meat and meat products, and how they score compared to products from other food categories:

*‘An unreasonable result of this scoring system is that healthy, nutritious and popular products such as cooked ham or turkey fillet get a D, which is worse or the same as unhealthy products like candies and desserts, chocolate pudding receiving a C.’* (Submission no. 3, Meat sector)

and for dairy products:

*‘[…] We believe it is unfortunate if ice lollies appear as an equal alternative to fruit yoghurt with muesli. […] Both ice lollies and muesli yoghurt can get a C.’* (Submission no. 8, Dairy sector)

Some actors also expressed concerns with Nutri-Score not differentiating between single products, portion sizes, and full meals, which may be confusing for consumers.

*‘It is even more challenging that the score does not differentiate between portion sizes, single products, and full meals. For example, you do not eat sandwich toppings separately, but in combination with several food items in a meal.’* (Submission no. 4, Meat sector)

One food system actor claimed that a continuous revision of the Nutri-Score is important to be able to adapt to future changes in the market.

### Arguments regarding the scientific evidence

Food system actors commented that there is a lack of evidence for using heme iron as an argument to reduce the protein score for red meat.

*‘Nutri-Score has included an upper limit of the number of points for protein that may be given to red meat with the argument that the content of heme iron may be harmful. This is a theory which is not evidence-based.’* (Submission no. 4, Meat sector)

The substantial evidence-base for the positive health effects of a high intake of whole grains was highlighted as an argument for including whole grain as a component in the Nutri-Score algorithm.

*‘In the NNR 2022 [Nordic Nutrition Recommendations 2023] chapter on grains, they conclude that there is convincing or probable evidence that a high intake of whole grains reduces the risk of colorectal cancer, cardiovascular diseases, and type 2 diabetes. Further, randomized controlled trials show that a high intake of whole grains is beneficial for blood pressure, cholesterol, and body weight. It should be considered whether it is possible to include whole grains in the Nutri-Score labeling system.’* (Submission no. 7, Grain sector)

### Policy-related issues

The food system actors were asked to evaluate Nutri-Score and alignment with Norwegian food-based dietary guidelines; however, they also reported on other broader topics. Political issues around Nutri-Score were mentioned, and actors referred to the opposition against Nutri-Score among European countries and the uncertain outcome of ongoing policy processes in the European Union. In light of these issues, they questioned the use of Nutri-Score as a basis for a scoring system in NewTools. They also referred critique related to unfavorable scoring of foods from local food production and local food culture. One response pointed to possible economical motives.

*‘At a European level, there has been opposition to Nutri-Score, as some local and traditional foods receive a poor score. The motives are economical. We believe that the content of the food and an optimal diet for public health should be the purpose of the score.’* (Submission no. 1, Civil society organization)

Actors representing the food industry criticized that Nutri-Score was inconsistent with Norwegian nutrition policies, in particular the criteria for the Keyhole label (26), which is owned by national health authorities, and the targets set in the partnership between the food industry and the health authorities to improve the diet of the population (31). These discrepancies were considered relevant for products in several categories, including meat, dairy, fats and oils, ready meals, and bread and cereals. Products that align with Keyhole criteria or partnership for a healthier diet targets may receive poor score with Nutri-Score due to its updated algorithm’s stringent criteria, especially concerning salt content.

*‘The food industry has in cooperation with the health authorities worked well and systematically to reduce salt in foods since 2015. For several products, the salt targets have been achieved. The results have been more Keyhole-labelled products and products in line with the Norwegian Directorate of Health’s dietary recommendations. […] It is most unfortunate that the industry’s work for healthier products will not be rewarded with Nutri-Score.’* (Submission no. 11, Producer of various food products)

The protein cap for red meat products was also reported as a reason for inconsistency between Nutri-Score and the Norwegian food-based dietary guidelines of choosing lean meat and meat products, where the protein content is often higher than the full fat options.

Products like regular and soft sweet whey cheeses were mentioned as examples of foods, which receive a poor score, despite being good iodine sources and recommended for children according to national guidelines for food and meals in kindergartens.

## Discussion

Food system actors, including food industry, consumers, and research/education, responded to this consultation about the performance of the updated Nutri-Score algorithms in a Norwegian setting. In total, they expressed a variety of responses relating to the updated Nutri-Score and discrepancies with Norwegian nutrition guidelines and policies. However, they provided only limited responses to national dietary challenges. Responses reflected the food system actors’ concerns related to different foods and nutrients, such as too much emphasis on salt; insufficient differentiation between full-fat and reduced fat alternatives of meat, sausages, cheese, and milk; several perceived unreasonable comparisons across food categories; and that Nutri-Score may stimulate to increased food processing. Food industry actors mainly focused on the calculation of specific components in the algorithms and misalignment between Nutri-Score and some national nutrition policies. The research and education institution was concerned with the lack of differentiation of some foods, for example, flours according to coarseness. Furthermore, there were mixed responses to the protein-cap on red meat and whether food processing level should be taken into consideration in the Nutri-Score algorithms.

In our previous evaluation of Nutri-Score in a Norwegian context (22), some minor inconsistencies between the Norwegian food-based dietary guidelines and the updated Nutri-Score were found, including inability to discriminate between regular and reduced-fat alternatives of cheese, cooking creams, and sausages, and between whole grain and refined pasta and rice. Food system actors in the present study pointed to several of the same inconsistencies, including the perception that the updated Nutri-Score does not align with the Norwegian food-based dietary guidelines for red meat, yogurt, cheese, milk, and whole grain. In the present study, some responders also described additional aspects that were not identified in the previously mentioned article (22), as discussed below.

Increased intake of fiber and whole grain products is recommended in the Norwegian food-based dietary guidelines (32). Food system actors in the current study mentioned that the fiber component in the updated Nutri-Score does not capture the wider range of fiber content both at the lower and higher ends of the scale. A wider scale would capture meaningful variation in fiber content in ready meals and in Norwegian bread and cereal products. However, one response mentioned that setting a lower threshold for fiber content to reward favorable points may incentivize the addition of pure fiber for the purpose of achieving a better Nutri-Score classification. The inclusion of whole grain in the Nutri-Score algorithm has been requested by different food system actors in European countries (33). However, as mentioned by the developers of Nutri-Score (10), there is no standardized definition of whole grain across Europe, making this a challenge.

The protein cap for red meat was introduced in the updated Nutri-Score food algorithm, limiting the favorable points for red meat products. This aligns with the newly published Nordic Nutrition Recommendations 2023 (34), which recommend to limit the intake of red meat (35). However, the imposition of this protein cap for red meat products was debated in the current study, with several food system actors expressing criticism while others supported the measure. Opinions about how the original Nutri-Score classify meat products with poor Nutri-Score classes have also been voiced in Spain (36), Italy (37), Hungary (20), and Poland (20), and these countries have requested certain modifications or exemptions for traditional products (20).

The Nutri-Score is primarily meant as an instrument for comparisons *within* food categories (10). However, criticism based on comparisons *across* food categories was reported in the current study. These included comparing red meat products to foods in different categories, such as junk food. For beverages, the findings suggested that the updated Nutri-Score might not adequately differentiate between the nutritional quality of beverages, such as milk versus artificially sweetened drinks, or fruit juices versus sodas. Spanish politicians have written to the European Commission to warn of the potential damage caused by the Nutri-Score to the well-known Iberian ham bellota, arguing that Nutri-Score indicates the ham having the same level of nutritional quality as junk food (36). This could also reflect questions relevant from a consumer perspective since it may not be obvious to consumers that the scoring is not meant for direct comparisons across food categories.

Similar to concerns raised in Europe (15), several food system actors in the current study argued that the updated Nutri-Score algorithms do not capture the level of processing, which could potentially stimulate food processing and unfairly reward, for example, highly processed plant-based alternatives to meat and dairy products. Researchers involved in the Nutri-Score development report that Nutri-Score provides information related to the nutritional composition of food, and that other health dimensions of food, such as processing, are not taken into account as this currently is impossible to scientifically encompass into a synthetic indicator (15). The concept of ultra-processed foods has recently received attention and been debated in Norwegian media (38, 39) due to the preparation of the Nordic Nutrition Recommendations published in June 2023 (35). This report did not specify a recommendation on ultra-processed foods due to limitations in evidence, as the current classification of ultra-processed foods does not add to the already existing food classification and recommendations in the Nordic Nutrition Recommendations (35). Nonetheless, Nutri-Score incorporating graphical information indicating ultra-processing to aid consumers in identifying the level of processing has been suggested (40).

Another finding in the current study was related to input on Nutri-Score’s lack of consistency with existing nutrition policies in Norway. These policies include the Nordic Keyhole and the public–private partnership for a healthier diet (31), both of which are supported by both authorities and the food industry. These policy measures involve voluntary actions to reduce the level of salt, sugars, and saturated fat in foods and diets, and both have received significant investment over several years. An EU-harmonized mandatory FOPNL system, if implemented as indicated in the Farm-to-fork strategy, will influence established Norwegian nutrition policy related to labeling and the Nordic Keyhole (26). If Nutri-Score becomes a contender in policy processes toward a new FOPNL in the EU, this scoring system will likely meet opposition due to perceived weaknesses in ranking food according to nutritional quality. Additionally, the Keyhole label, which is well established and considered simpler to consumers by some, may remain preferred despite having also faced criticism. Mandatory FOPNL was recently recommended by an expert group as one of the five key cost-effective nutrition policy recommendations for the Norwegian government to consider, and the Nutri-Score was an example of one FOPNL system that could be assessed (41).

## Strengths and limitations

The strength of this study was the involvement of a variety of actors in the NewTools-project as representatives for the Norwegian food system. Many of the actors providing feedback have in-depth knowledge about their products, which is beneficial when testing nutrient profiling models. The findings add to the previous evaluation of Nutri-Score in a Norwegian setting performed by researchers in NewTools (22) by capturing perspectives of food system actors with in-depth knowledge of foods and food production, processing, and manufacturing. This insight could contribute with valuable input for future work on nutrient profiling systems and contribute to the NewTools-project. Within NewTools, a primary goal is to develop a scoring system to assess the nutritional quality of foods, which involves identifying challenges and potentially making revisions. Another strength is using an established framework for collaboration in the NewTools-project (25) to ensure scientific integrity and help prevent and manage conflict of interests as food system actors involved in NewTools include actors with vested interests in food profiling systems.

A limitation is, however, that not all relevant stakeholders across the Norwegian food system are involved in the NewTools-project, which may result in certain aspects not being adequately addressed. A significant proportion (70%) of the invited actors refrained from responding in the consultations. One potential explanation could be attributed to their primary interest in the environmental sustainability part of the project or not wanting to provide an official assessment of Nutri-Score. Five of the 13 submissions were from actors representing the meat and dairy sector and farmers, which may indicate that these actors perceived Nutri-Score as most challenging due to poor score for some meat and dairy products. Only one non-governmental organization and one research and education institution responded, which means that the perspectives of public health advocacy and scientific scrutiny of the Nutri-Score may be under-represented in the current study. In the information meetings about the consultations, there were actors represented who did not submit responses, including partners from the fruit and vegetable sector, the seafood sector, and retail. This may indicate that some judged the updated Nutri-Score as satisfactory or considering that providing a response was not relevant. For example, fruit or vegetable producers may not prioritize responding as such products mainly achieve high Nutri-Score grades. On the other hand, it may be time-consuming to provide detailed responses, and the invited food system actors could have lacked time or resources to send a response. Therefore, this study may have missed perspectives from relevant actors in the food system. However, the composition of food system actors providing responses in the current study is quite similar to the composition of stakeholders in the consultation the Scientific Committee of the Nutri-Score conducted before the update of the algorithms, where a limited number of responses were received from consumer groups, non-governmental organizations, and nutrition-related professional groups, and most responses were from the agro-industry sector and individual producers or manufacturers (33).

## Conclusion

The current study described a range of concerns with the updated Nutri-Score algorithms among actors from the Norwegian food system. This included specific nutritional concerns, unreasonable comparisons across food categories, and inconsistency between Nutri-Score and certain established nutrition policies. These responses contribute with insight and add useful knowledge when evaluating Nutri-Score’s alignment with dietary guidelines and nutrition policies in Norway. However, the results should be interpreted with caution as some perspectives from the Norwegian food system may be missing.

## Supplementary Material








