# Supplementary material for: Responses to the updated Nutri-Score algorithms in Norway: A qualitative study among food system actors in the NewTools-project
Source: Food Nutr Res. 2024 Oct 1;68:10.29219/fnr.v68.10254. doi: 10.29219/fnr.v68.10254 (PMC11457909; doi:10.29219/fnr.v68.10254)
Supplement: Supplementary file 1 [file FNR-68-10254-s1.pdf]

Methods

The publicly available Norwegian food database was used to describe Nutri-Score in a Norwegian setting. Nutri-Score was calculated as described in the updated algorithm report (2022) and details about the calculation can be read in the report (1). The Nutri-Score beverage algorithm has not been updated, so all beverages and foods ineligible for Nutri-Score were excluded from the dataset. The food composition database (2021) was downloaded from Matportalen.no (2). Nutri-Score is presented for each food item in the Excel-file. Nutri-Score for main categories of foods and for more specific categories of foods are presented below. Information about which products that were included in the various food categories are shown in log-files. We present the distribution of Nutri-Score in tables and figures, where the figures show the distribution of Nutri-Score using violin- and boxplots. Violin plots show the continuous distribution of the foods in each category, while the box in a boxplot shows summary statistics using the median (50th percentile) and interquartiles (25th percentile and 75th percentile).

Nutri-Score in a Norwegian setting by main categories of food

Table 1. Distribution of Nutri-Score by main categories of food.

	**Nutri-Score**					**Total**
	**A**	**B**	**C**	**D**	**E**	
Milk products	7<br>6.8	7<br>6.8	14<br>13.6	54<br>52.4	21<br>20.4	103<br>100.0
Eggs	3<br>60.0	0<br>0.0	0<br>0.0	2<br>40.0	0<br>0.0	5<br>100.0
Poultry and meat*	56<br>25.0	12<br>5.4	42<br>18.8	77<br>34.4	37<br>16.5	224<br>100.0
Fish and shellfish*	80<br>50.3	19<br>12.0	30<br>18.9	16<br>10.1	14<br>8.8	159<br>100.0
Cereals and baked goods	68<br>21.3	41<br>12.9	76<br>23.8	50<br>15.7	84<br>26.3	319<br>100.0
Potatoes, vegetables, fruits and berries*	213<br>72.2	16<br>5.4	40<br>13.6	22<br>7.5	4<br>1.4	295<br>100.0
Sugar and sweet products	1<br>2.2	0<br>0.0	0<br>0.0	7<br>15.2	38<br>82.6	46<br>100.0
Margarine, butter, cooking oil, etc.	0<br>0.0	11<br>16.4	19<br>28.4	18<br>26.9	19<br>28.4	67<br>100.0
Nuts and seeds	16<br>45.7	7<br>20.0	8<br>22.9	4<br>11.4	0<br>0.0	35<br>100.0
Other dishes, products and ingredients	38<br>21.2	22<br>12.3	54<br>30.2	39<br>21.8	26<br>14.6	179<br>100.0
Total	482<br>33.7	135<br>9.4	283<br>19.8	289<br>20.2	243<br>17.0	1432<br>100.0

First row show frequencies (n) and second row show percentages (%), for each category.
*Include products thereof and dishes.

See the following file for a list of all foods included in the different food categories in the table above: "Content main food categories.log".

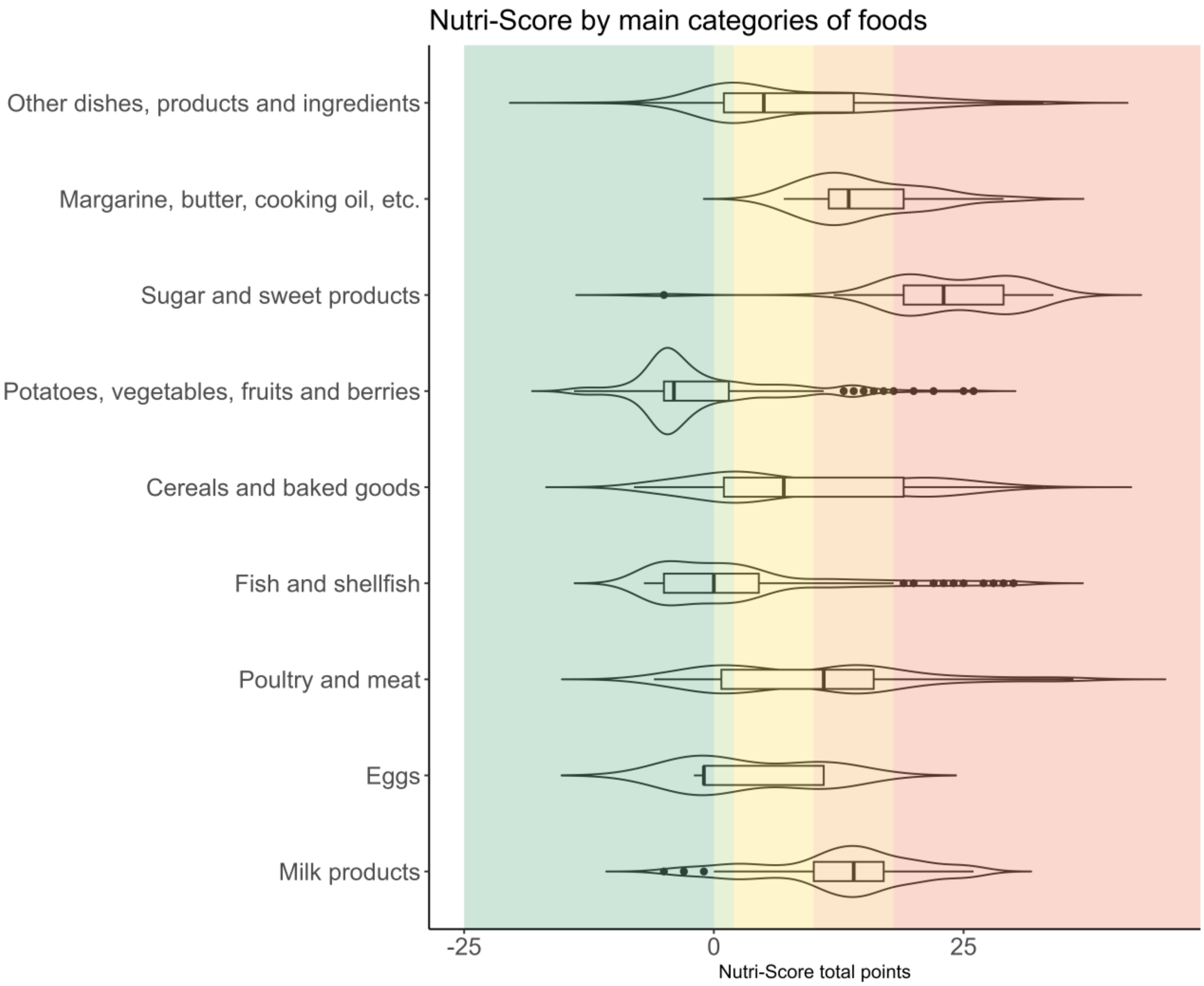

Figure 1. Distribution of Nutri-Score by main categories of general foods, shown by violin- and boxplots. Dark green background color indicates Nutri-Score class A, light green indicates B, yellow indicates C, light orange indicates D and dark orange indicates Nutri-Score E. Milk products include yogurts and cheeses, see "Content main food categories.log" for details.

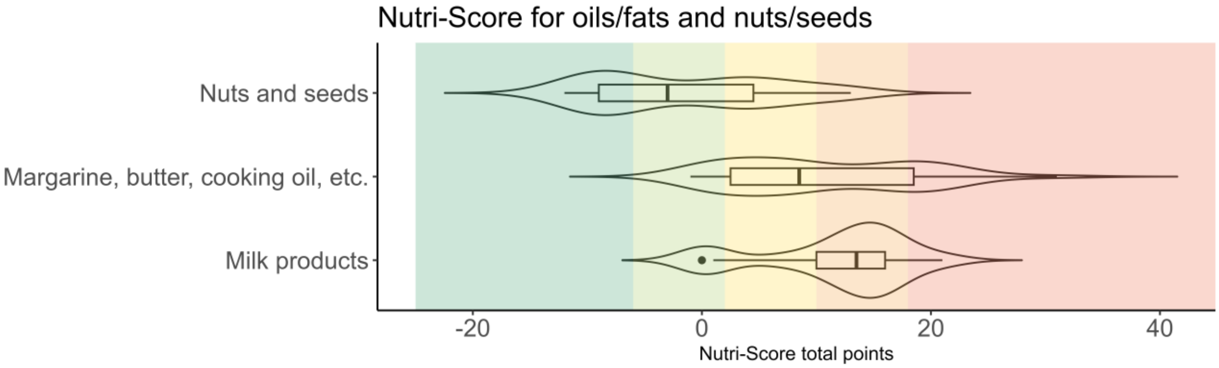

Figure 2. Distribution of Nutri-Score by main categories of fats, oils, nuts and seeds, shown by violin- and boxplots. Dark green color on background indicates Nutri-Score A, light green indicates B, yellow indicates C, light orange indicates D and dark orange indicates Nutri-Score E. The category dairy products include cream, sour cream, crème fraise, etc., see "Content main food categories.log" for details.

Nutri-Score in a Norwegian setting by specific categories of foods

Table 2. Distribution of Nutri-Score by specific categories of foods.

	Nutri-Score					Total
	**A**	**B**	**C**	**D**	**E**	
Yoghurt/quark	5	3	4	0	0	12
	41.7	25.0	33.3	0.0	0.0	100,0
Cream, sour cream, cream substitutes	0	3	1	11	1	16
	0.0	18.8	6.3	68.8	6.3	100.0
Cheese	2	0	9	42	19	72
	2.8	0.0	12.5	58.3	26.4	100.0
Eggs	3	0	0	2	0	5
	60.0	0.0	0.0	40.0	0.0	100.0
Poultry and poultry products	20	1	4	13	0	38
	52.6	2.6	10.5	34.2	0.0	100.0
Red meat and meat products	23	7	26	56	36	148
	15.5	4.7	17.6	37.8	24.3	100.0
Other meats	9	1	0	3	1	14
	64.3	7.1	0.0	21.4	7.1	100.0
Dishes with poultry or meat	4	3	12	5	0	24
	16.7	12.5	50.0	20.8	0.0	100.0
Fish, fish products and shellfish	64	12	24	16	14	130
	49.2	9.2	18.5	12.3	10.8	100.0
Dishes with fish, shellfish, etc.	16	7	6	0	0	29
	55.2	24.1	20.7	0.0	0.0	100.0
Flour	23	2	8	2	0	35
	65.7	5.7	22.9	5.7	0.0	100.0
Grain, rice, pasta	19	14	5	2	0	40
	47.5	35.0	12.5	5.0	0.0	100.0
Breakfast cereals	8	6	12	4	1	31
	25.8	19.4	38.7	12.9	3.2	100.0
Breads	10	14	22	5	1	52
	19.2	26.9	42.3	9.6	1.9	100.0
Crispbread, crackers, etc.	8	3	16	2	3	32
	25.0	9.4	50.0	6.3	9.4	100.0
Cookies, sweet biscuits, rusks	0	1	0	5	26	32
	0.0	3.1	0.0	15.6	81.3	100.0
Yeast cake, griddle cake, waffles, etc.	0	1	11	14	4	30
	0.0	3.3	36.7	46.7	13.3	100.0
Other cakes etc.	0	0	2	16	49	67
	0.0	0.0	3.0	23.9	73.1	100.0
Nuts and seeds	16	7	8	4	0	35
	45.7	20.0	22.9	11.4	0.0	100.0
Potatoes and potato products	4	5	5	0	0	14
	28.6	35.7	35.7	0.0	0.0	100.0
Vegetables	108	4	12	5	4	133
	81.2	3.0	9.0	3.8	3.0	100.0
Legumes	27	0	0	0	0	27

	100.0	0.0	0.0	0.0	0.0	100.0
Fruits and berries, incl. jam	74	7	23	17	0	121
	61.2	5.8	19.0	14.1	0.0	100.0
Sugar, honey and sweet spreads	0	0	0	2	10	12
	0.0	0.0	0.0	16.7	83.3	100.0
Chocolate and other sweets	1	0	0	5	28	34
	2.9	0.0	0.0	14.7	82.4	100.0
Margarine and butter	0	0	9	6	9	24
	0.0	0.0	37.5	25.0	37.5	100.0
Cooking oil, frying fat, etc.	0	11	4	1	3	19
	0.0	57.9	21.1	5.3	15.8	100.0
Mayonnaise, dressing, etc.	0	0	6	12	7	25
	0.0	0.0	24.0	48.0	28.0	100.0
Pizza, pie, tacos, ready meals, etc.	6	3	8	9	0	26
	23.1	11.5	30.8	34.6	0.0	100.0
Porridge	3	2	5	1	0	11
	27.3	18.2	45.5	9.1	0.0	100.0
Soup, sauce/gravy, stew base	7	4	12	2	0	25
	28.0	16.0	48.0	8.0	0.0	100.0
Dessert, ice cream, etc.	2	4	16	14	13	49
	4.1	8.2	32.7	28.6	26.5	100.0
Snacks	0	1	4	6	4	15
	0.0	6.7	26.7	40.0	26.7	100.0
Vegetarian products and dishes	20	8	7	2	2	39
	51.3	20.5	18.0	5.1	5.1	100.0
Miscellaneous ingredients	0	1	2	4	3	10
	0.0	10.0	20.0	40.0	30.0	100.0
Powder base, dry	0	0	0	1	5	6
	0.0	0.0	0.0	16.7	83.3	100.0
Total	482	135	283	289	243	1432
	33.7	9.4	19.8	20.2	17.0	100.0

First row show frequencies (n) and second row show percentages (%), for each category.

See the following file for a list of all foods included in the different food categories in the table above: "Content specific food categories.log".

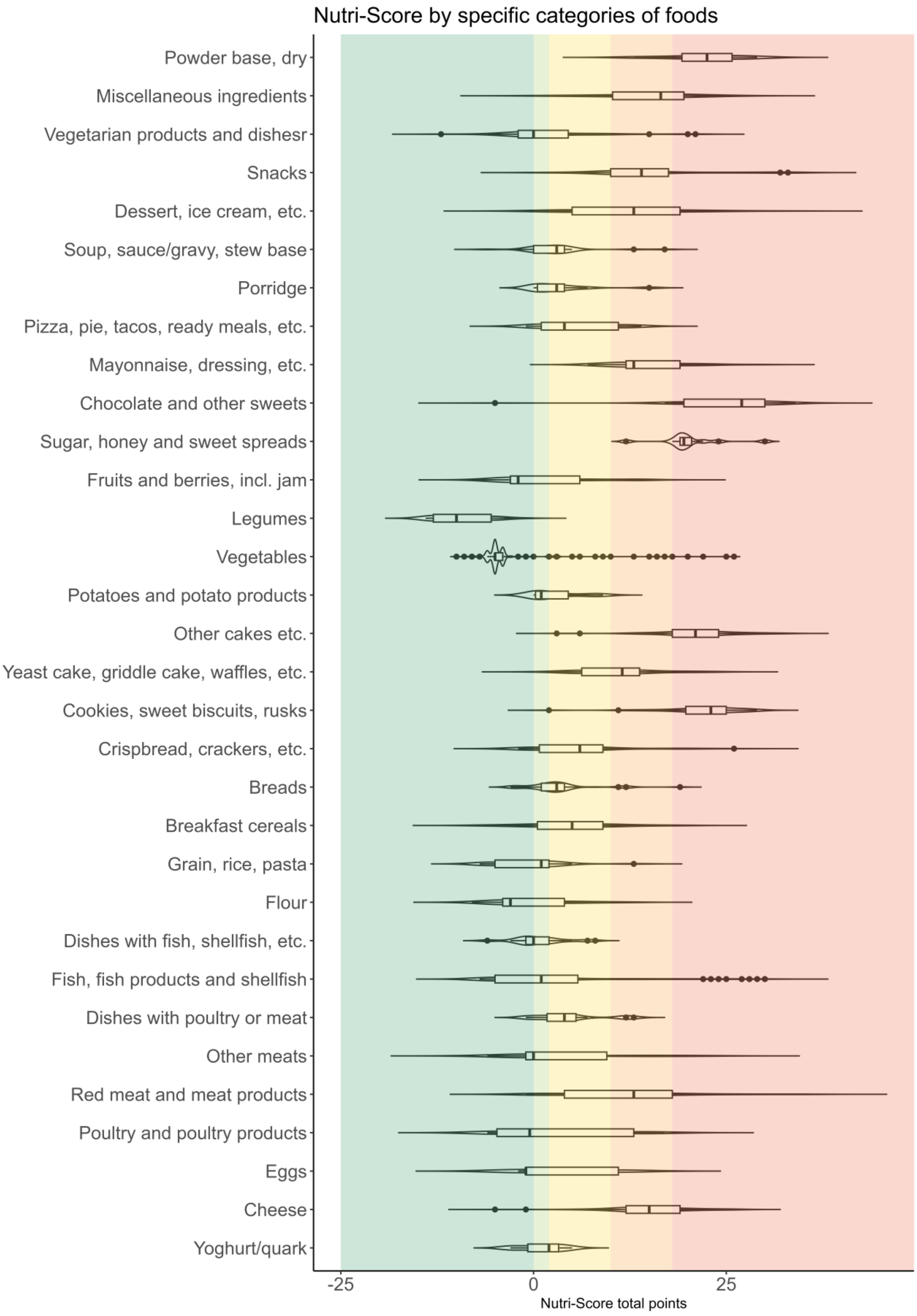

Nutri-Score by specific categories of foods
Powder base, dry
Miscellaneous ingredients
Vegetarian products and dishesr
Snacks
Dessert, ice cream, etc.
Soup, sauce/gravy, stew base
Porridge
Pizza, pie, tacos, ready meals, etc.
Mayonnaise, dressing, etc.
Chocolate and other sweets
Sugar, honey and sweet spreads
Fruits and berries, incl. jam
Legumes
Vegetables
Potatoes and potato products
Other cakes etc.
Yeast cake, griddle cake, waffles, etc.
Cookies, sweet biscuits, rusks
Crispbread, crackers, etc.
Breads
Breakfast cereals
Grain, rice, pasta
Flour
Dishes with fish, shellfish, etc.
Fish, fish products and shellfish
Dishes with poultry or meat
Other meats
Red meat and meat products
Poultry and poultry products
Eggs
Cheese
Yoghurt/quark
-25
0
25
Nutri-Score total points

Figure 3. Distribution of Nutri-Score by specific categories of foods, shown by violin- and boxplots.
Dark green background color indicates Nutri-Score class A, light green indicates B, yellow indicates C, light orange indicates D and dark orange indicates Nutri-Score E. Milk products include yogurts and cheeses, see "Content specific food categories.log" for details.

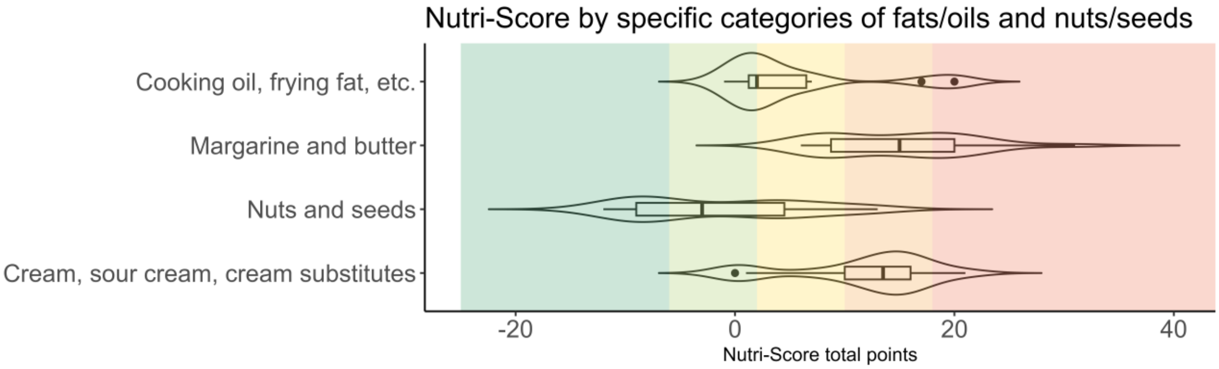

Figure 4. Distribution of Nutri-Score by more specific categories of fats, oils, nuts and seeds, shown by violin- and boxplots.
Dark green background color indicates Nutri-Score class A, light green indicates B, yellow indicates C, light orange indicates D and dark orange indicates Nutri-Score E. See "Content specific food categories.log" for details.

References

1. The Scientific Committee of the Nutri-Score. Update of the Nutri-Score algorithm - Update report from the Scientific Committee of the Nutri-Score 2022. 2022. Tilgjengelig fra: https://www.bmel.de/SharedDocs/Downloads/EN/_Food-and-Nutrition/nutri-score-update-algorithm.pdf?__blob=publicationFile&v=2
2. Norwegian Food Composition Database 2021. Norwegian Food Safety Authority. Downloaded July 4. 2022 from www.matvaretabellen.no
