# Supplementary material for: Responses to the updated Nutri-Score algorithms in Norway: A qualitative study among food system actors in the NewTools-project
Source: Food Nutr Res. 2024 Oct 1;68:10.29219/fnr.v68.10254. doi: 10.29219/fnr.v68.10254 (PMC11457909; doi:10.29219/fnr.v68.10254)
Supplement: Supplementary file 2 [file FNR-68-10254-s2.pdf]

Methods

The publicly available Norwegian food database was used to describe Nutri-Score in a Norwegian setting. Nutri-Score was calculated as described in the updated algorithm report for beverages (2023) and details about the calculation can be read in the report (1). The updated algorithm for foods has already had a consultation round. Beverages ineligible for Nutri-Score were excluded from the dataset, such as homemade smoothies. The food composition database (2021) was downloaded from Matportalen.no (2). Nutri-Score is presented for each food item in the Excel-file.

Nutri-Score for main categories of beverages and for more specific categories of beverages are presented below. Information about which products that were included in the various beverage categories are shown in log-files. We present the distribution of Nutri-Score in tables and figures. The figures show the distribution of Nutri-Score using boxplots and dots. The box in a boxplot shows summary statistics using the median (50th percentile) and interquartiles (25th percentile and 75th percentile). The dots indicate single beverage items. In the updated algorithm on beverages, plain water gets Nutri-Score class A, as the only beverage that can get this. Water is shown in the figures, despite the fact that the Nutri-Score total points for water cannot be calculated.

Nutri-Score in a Norwegian setting by main categories of beverages

Table 1. Distribution of Nutri-Score by main categories of beverages.

	Nutri-Score					Total
	**A**	**B**	**C**	**D**	**E**	
Milk and milk-based drinks	0<br>0.0	24<br>60.0	13<br>32.5	1<br>2.5	2<br>5.0	40<br>100.0
Water, coffee, tea	3<br>7.9	27<br>71.1	6<br>15.8	2<br>5.3	0<br>0.0	38<br>100.0
Juices, squash, soda, etc.	0<br>0.0	5<br>8.3	18<br>30.0	19<br>31.7	18<br>30.0	60<br>100.0
Total	3<br>2.2	56<br>40.6	37<br>26.8	22<br>15.9	20<br>14.5	138<br>100.0

First row show frequencies (n) and second row show percentages (%), for each category.

See the following file for a list of all beverages included in the different categories in the table above: "Content main beverage categories.log".

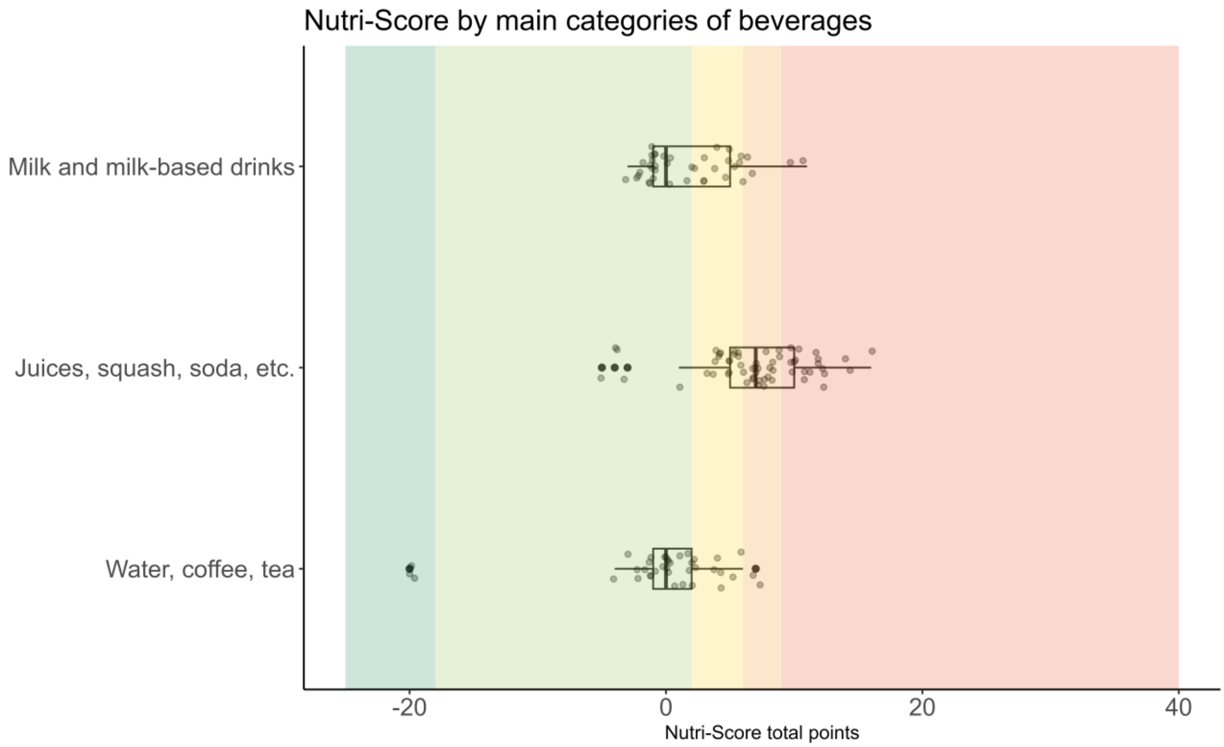

Figure 1. Distribution of Nutri-Score by main categories of beverages, shown by boxplots and dots.
Dark green background color indicates Nutri-Score class A (plain water only), light green indicates B, yellow indicates C, light orange indicates D and dark orange indicates Nutri-Score E. Light-gray dots illustrate individual beverage items. See "Content main beverage categories.log" for details on beverages in the categories.

Nutri-Score in the Norwegian setting by more specific categories of beverages

Table 2. Distribution of Nutri-Score by more specific categories of beverages.

	Nutri-Score					Total
	**A**	**B**	**C**	**D**	**E**	
Plain water	3	0	0	0	0	3
	100.0	0.0	0.0	0.0	0.0	100.0
Juices, squash, etc.	0	1	10	13	9	33
	0.0	3.0	30.3	39.4	27.3	100.0
Coffee, tea	0	27	6	2	0	35
	0.0	77.1	17.1	5.7	0.0	100.0
Soda, etc.	0	0	5	0	7	12
	0.0	0.0	41.7	0.0	58.3	100.0
Milk and plant-based	0	28	16	7	4	55
milk	0.0	50.9	29.1	12.7	7.3	100.0
Total	3	56	37	22	20	138
	2.2	40.6	26.8	15.9	14.5	100.0

First row show frequencies (n) and second row show percentages (%), for each category.

See the following file for a list of all beverages included in the different categories in the table above: "Content specific beverage categories.log".

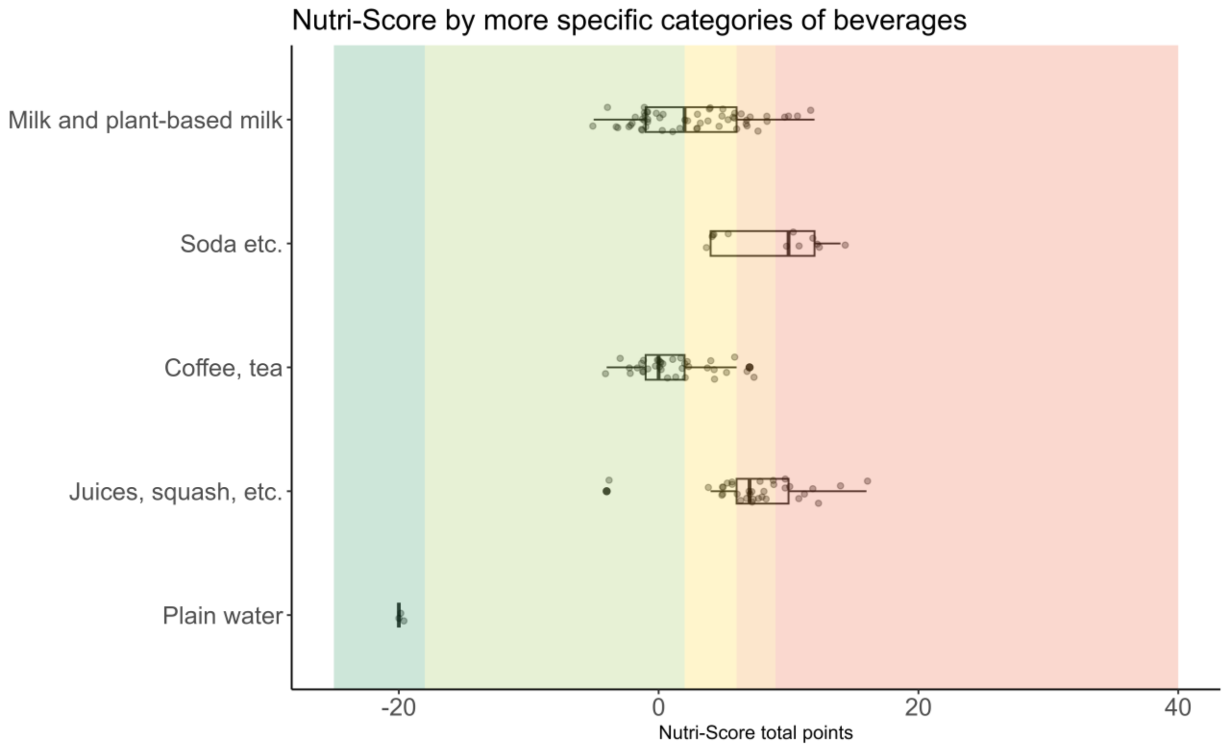

Figure 2. Distribution of Nutri-Score by more specific categories of beverages, by boxplots and dots.

Dark green background color indicates Nutri-Score class A (plain water only), light green indicates B, yellow indicates C, light orange indicates D and dark orange indicates Nutri-Score E. Light-gray dots illustrate individual beverage items. See " Content specific beverage categories.log" for details on beverages in the categories.

References

1. The Scientific Committee of the Nutri-Score. Update of the Nutri-Score algorithm for beverages – Second update report from the Scientific Committee of the Nutri-Score V2 - 2023. 2023. Tilgjengelig fra: https://sante.gouv.fr/IMG/pdf/update_report_beverages_31_01_2023-voted.pdf
2. Norwegian Food Composition Database 2021. Norwegian Food Safety Authority. Downloaded July 4. 2022 from www.matvaretabellen.no
